# The Impact of Preoperative Risk Factors on Delayed Discharge in Day Surgery: A Meta-Analysis

**DOI:** 10.3390/healthcare13020104

**Published:** 2025-01-08

**Authors:** Hanqing Zhang, Xinglian Gao, Zhen Chen

**Affiliations:** 1 Union Hospital, Tongji Medical College, Huazhong University of Science and Technology, Wuhan 430000, China; m202375993@hust.edu.cn; 2 School of Nursing, Tongji Medical College, Huazhong University of Science and Technology, Wuhan 430030, China; 3 Eye Center, Renmin Hospital of Wuhan University, Wuhan 430060, China

**Keywords:** day surgery, preoperative risk factors, meta-analysis, delayed discharge

## Abstract

Objective: This study aims to evaluate and identify the main preoperative risk factors affecting the timely discharge of day surgery patients, offering evidence to enhance preoperative assessments and minimize delayed discharge. Background: With the widespread adoption of day surgery in global healthcare systems, ensuring timely discharge of patients post-surgery has become a critical challenge. Numerous studies have explored various preoperative risk factors influencing delayed discharge. This meta-analysis integrates existing evidence to clarify the primary preoperative risk factors. Methods: A systematic search was conducted across the PubMed, CINAHL, Scopus, Web of Science, Embase, Cochrane Library, and CNKI databases, including all clinical studies on preoperative risk factors for day surgery published until 15 October 2024. A systematic review and random effects model were employed to aggregate data and estimate the main preoperative risk factors for day surgery. Results: A total of nine studies involving 41,458 patients were included. The analysis revealed statistically significant differences in the following preoperative risk factors: age (MD = 1.33, 95% CI: 0.73–1.93, *p* < 0.0001), body mass index (BMI) (MD = 0.69, 95% CI: 0.18–1.20, *p* = 0.008), the presence of chronic comorbidities (OR = 3.62, 95% CI: 2.93–4.46, *p* < 0.00001), the type of anesthesia (OR = 15.89, 95% CI: 7.07–35.69, *p* < 0.00001), a history of cardiac disease (OR = 2.46, 95% CI: 1.71–3.53, *p* < 0.00001), gender (OR = 3.18, 95% CI: 2.03–4.99, *p* < 0.00001), the expected duration of surgery (MD = 0.18, 95% CI: 0.15–0.20, *p* < 0.00001), complex procedures (OR = 1.78, 95% CI: 1.47–2.16, *p* < 0.00001), a lack of social family support (OR = 2.42, 95% CI: 1.60–3.67, *p* < 0.0001), and inadequate preoperative assessment (OR = 3.64, 95% CI: 2.06–6.41, *p* < 0.00001). There were no statistically significant differences between the delayed discharge group and the non-delayed discharge group in terms of the American Society of Anesthesiologists (ASA) classification (*p* = 1.00) and preoperative anxiety (*p* = 0.08). Conclusion: This study identifies the primary preoperative risk factors for delayed discharge in day surgery, including age, high BMI, the presence of chronic comorbidities, the type of anesthesia, a history of cardiac disease, gender, the duration of surgery, the complexity of the procedure, a lack of social family support, and inadequate preoperative assessment. These findings provide a reference for preoperative assessment, highlighting the need for clinical attention to these high-risk groups during preoperative screening and management to reduce the likelihood of delayed discharge and enhance surgical safety and success rates.

## 1. Introduction

With advancements in global healthcare systems, day surgery has emerged as a common model of medical service, offering significant advantages such as reduced hospital stays, lower healthcare costs, decreased risk of postoperative infections, and improved utilization of hospital beds [1,2]. Preoperative risk assessment is a critical step in determining whether day surgery can proceed smoothly [3]. Through this assessment, clinicians can accurately identify and manage various risk factors that may impact surgical success, such as the patient’s age, BMI, and history of previous illnesses [4,5]. Given that day surgery patients require prompt discharge after the procedure, the identification and management of preoperative risk factors are particularly important. Failure to adequately consider these factors may lead to postoperative complications and an increased risk of delayed discharge, and, potentially, affect long-term patient outcomes [6].

Although existing studies have explored various preoperative risk factors, inconsistent conclusions arise due to differences in research methodologies, sample characteristics, and analytical strategies [7]. For instance, regarding the impact of age on day surgery outcomes, some studies indicate that advanced age is a major risk factor, while others fail to find a significant association [8]. Furthermore, factors such as a high BMI, a history of cardiovascular disease, and the presence of chronic comorbidities are considered significant risk factors in some studies [9], yet their generalizability across different surgical types and populations has not been thoroughly validated [9,10].

Age and BMI are critical factors influencing surgical recovery, particularly among elderly and obese patients who are more prone to postoperative complications such as delayed wound healing or respiratory issues. Additionally, patients undergoing complex surgeries, such as those requiring general anesthesia and extended operative times, typically experience longer postoperative recovery periods [11], whereas those undergoing simpler procedures with local anesthesia tend to recover more quickly [12]. These differences highlight the importance of preoperative assessment, especially for high-risk patients, such as the elderly, obese, or those scheduled for complex surgeries. Personalized evaluation and management can effectively reduce the risk of delayed discharge in these populations.

Thus, the purpose of this meta-analysis is to systematically identify preoperative risk factors significantly associated with delayed discharge in day surgery [13], and to clarify the roles of various preoperative risk factors in day surgery. The findings aim to provide scientific evidence to assist clinicians in optimizing decision-making during preoperative assessment and management, ultimately enhancing healthcare quality, increasing surgical success rates, and improving patient satisfaction.

## 2. Methods

### 2.1. Registration ID

The protocol for this review has been registered with the International Prospective Register of Systematic Reviews (PROSPERO), registration number CRD42024597953. This study adheres to the Preferred Reporting Items for Systematic Reviews and Meta-Analyses (PRISMA) statement and the Meta-analysis of Observational Studies in Epidemiology (MOOSE) guidelines.

### 2.2. Search Strategy

Literature retrieval was conducted by two authors independently using computer-based searches across several databases. The search timeframe was set from the inception of the databases until 15 October 2024, focusing on published articles regarding the impact of preoperative risk factors on delayed discharge in day surgery. A systematic search was performed across PubMed, CINAHL, Scopus, Web of Science, Embase, Cochrane Library, and CNKI electronic databases. The terms “Preoperative risk factors” OR “Preoperative assessment” OR “Preoperative evaluation” OR “Preoperative factors” OR “Pre-surgical risk factors” OR “Pre-surgical assessment” OR “Pre-surgical evaluation” AND “Day surgery” OR “Ambulatory surgery” OR “Same-day surgery” OR “Day-case surgery” were flexibly combined using Medical Subject Headings (MeSH) or free-text terms. There were no restrictions based on publication date, geographical location, or study design. However, the search was limited to studies published in English. The reference lists of included articles were also manually reviewed to capture all potentially relevant studies.

### 2.3. Inclusion and Exclusion Criteria

Inclusion criteria were as follows: (1) Study population: patients undergoing day surgery; (2) Study type: cohort and case-control studies on preoperative risk factors for day surgery; (3) Outcome measures: age, BMI, ASA classification, history of cardiac disease, type of anesthesia, presence of chronic comorbidities, duration of surgery, type of surgery, gender, preoperative anxiety, etc.; and (4) Data availability: provision of mean difference (MD), odds ratios (ORs), 95% confidence intervals (CIs), and other statistical data. Exclusion criteria were as follows: (1) Study type: basic clinical studies, editorials, animal studies, literature reviews, case reports, retracted articles, and conference abstracts; (2) Literature content: incomplete data, unavailable data, statistical errors, erroneous citations, and duplicate publications; (3) Access: unavailable sources. To ensure consistency in data quality, the following studies were excluded: (1) those reporting only qualitative descriptions without quantitative statistical data; (2) those with insufficient sample sizes that may introduce bias; and (3) those unable to provide key statistical parameters.

### 2.4. Data Extraction and Collection

Based on the inclusion and exclusion criteria, the two authors screened 46,086 articles. They organized the literature using EndNote 20 software, checked the titles, and independently read the titles, abstracts, and full texts after removing duplicates to extract data, with accuracy checked by a third independent author. In cases of disagreement, a senior author made the final decision. The following data points were extracted from each article: basic information (first author name, publication year, and study country); study design (study type, sample size, and inclusion and exclusion criteria); patient characteristics (age, gender, and BMI); surgical and anesthesia information (type of surgery, duration of surgery, and type of anesthesia); types of preoperative risk factors (age, BMI, comorbidities, history of cardiovascular disease, type of anesthesia, etc.); and the association between preoperative risk factors and delayed discharge in day surgery. If available, adjusted odds ratios (ORs) and their 95% confidence intervals (CIs) from multivariable regression analyses were also extracted, along with the variables included in the multivariable models for each study. Corresponding study authors were contacted to clarify any unclear or missing data. Ultimately, nine articles were included. In addition, preoperative anxiety was included as a major preoperative risk factor in the analysis. Although some studies did not show statistical significance, preoperative anxiety is closely associated with the rate of postoperative recovery. Therefore, studies containing relevant quantitative data were included to ensure the reliability of the data analysis.

### 2.5. Quality Assessment

The quality of the included studies was independently assessed by two authors. Cohort and case-control studies were evaluated using the Newcastle–Ottawa Scale (NOS). This scale specifically divides into three areas: (1) selection bias (including four sub-items), (2) comparability bias (including one sub-item), and (3) outcomes (including three sub-items). Articles meeting the criteria for the “comparability bias” item received 2 points, while the other sub-items received 1 point each, for a total score of 9 points. The protocol was interpreted based on established classifications: low quality (0–3 points), moderate quality (4–5 points), and high quality (6–9 points). The two authors cross-checked the quality assessments; if there was a disagreement, a third author facilitated a discussion to reach a consensus.

### 2.6. Data Analysis

Meta-analysis in this study was conducted using Review Manager software (RevMan 5.4) provided by Cochrane. For continuous outcome data, mean differences (MDs) or standardized mean differences (SMDs) were used as the primary effect size measures. For binary outcome data, odds ratios (ORs) or risk ratios (RRs) were used as the primary effect size measures, and their 95% confidence intervals (CIs) were calculated. The heterogeneity among the included studies was assessed using the I^2^ statistic. When I^2^ > 50%, it indicated considerable heterogeneity among the studies, necessitating subgroup and sensitivity analyses to identify and eliminate significant heterogeneity. When I^2^ < 50%, low heterogeneity among the studies was indicated, and a fixed effects model (FEM) was used for analysis. A *p* value < 0.05 was generally considered statistically significant. In the preliminary analysis, if some studies have small sample sizes or inconsistent data quality, significant heterogeneity may exist. Sensitivity analysis will be performed to gradually exclude individual studies with high heterogeneity. Meanwhile, this study employed the Benjamini–Hochberg correction method to control the false discovery rate (FDR) in multiple testing, ensuring the robustness and reliability of the statistical results.

## 3. Results

### 3.1. Literature Screening

**Figure 1:**
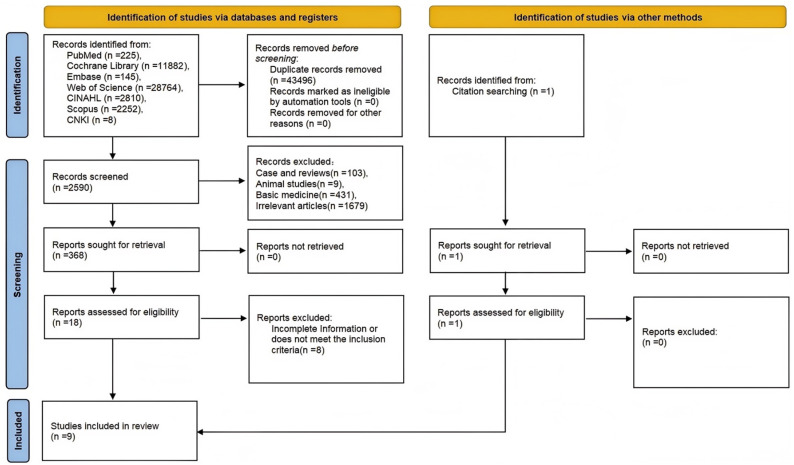
Flowchart of literature screening.

According to the established search strategy, a total of 48,086 articles were identified, and after review, 2590 articles were retained. Following a careful examination of the titles and abstracts, inclusion and exclusion criteria were established. After thoroughly reading the full texts, a total of nine articles were ultimately selected [2,5,6,7,8,14,15,16,17]. The screening process is illustrated in Figure 1.

### 3.2. Basic Characteristics of Included Studies

This meta-analysis included nine studies published between 2012 and 2022, comprising a total of 41,458 patients, with eight studies being retrospective, and the remaining one being a prospective cohort study. Among all included studies, the following characteristics were reported: Age was provided in all nine articles [2,5,6,7,8,14,15,16,17], ASA classification was reported in three articles [5,6,15], a history of cardiac disease was provided in five articles [2,5,6,8,16], chronic comorbidities were reported in eight articles [2,5,6,7,8,14,15,16], BMI was provided in five articles [5,6,7,8,15], the duration of surgery was reported in six articles [5,7,8,15,16,17], gender was provided in five articles [5,6,8,16,17], preoperative anxiety was reported in three articles [7,16,17], preoperative assessment was provided in six articles [2,5,8,14,16,17], the type of anesthesia was reported in six articles [2,5,6,7,8,14], the type of surgery was provided in seven articles [5,6,7,8,14,15,17], and a lack of social and family support was reported in seven articles [2,5,6,8,14,15,16]. The characteristics of the included studies are summarized in Table 1.

### 3.3. Quality Assessment of Included Studies

Our analysis included seven RCSs [2,5,7,8,14,16,17], one PCS [15], and one RCCS [6]. The assessment method employed was the NOS scoring. The evaluation results are presented in Table 2.

### 3.4. Meta-Analysis

This meta-analysis summarizes the key risk factors and their statistical outcomes in this study, including the OR/MD, 95% CI, *p*-value, and I^2^. These factors exhibit significant statistical and clinical relevance in delayed discharge, as detailed in Table 3.

After applying the Benjamini–Hochberg correction, the following risk factors remained statistically significant (*p* < 0.05): type of anesthesia (OR = 15.89, adjusted *p* < 0.00001), chronic comorbidities (OR = 3.62, adjusted *p* < 0.00001), history of cardiac disease (OR = 2.46, adjusted *p* < 0.00001), lack of social or family support (OR = 2.42, adjusted *p* < 0.0001), preoperative evaluation (OR = 3.64, adjusted *p* < 0.00001), type of surgery (OR = 1.78, adjusted *p* < 0.00001), age (MD = 1.33, adjusted *p* < 0.0001), gender (OR = 3.18, adjusted *p* < 0.00001), surgical duration (MD = 0.18, adjusted *p* < 0.00001), and BMI (MD = 0.69, adjusted *p* < 0.01).

**Figure 2:**
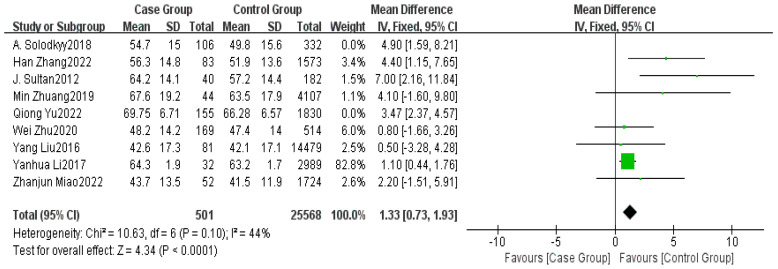
Forest plot of age. The *p*-values have been adjusted using the Benjamini–Hochberg correction, and significant factors have been marked accordingly. Green dot: Confidence interval endpoints of individual study effect sizes. Green square: Study effect sizes, with size representing weight. Black diamond: Overall effect size, showing weighted average and total confidence interval [2,5,6,7,8,14,15,16,17].

Nine studies provided data on age [2,5,6,7,8,14,15,16,17]. The meta-analysis indicated high heterogeneity among the studies (I^2^ = 68%). Sensitivity analysis revealed that excluding two studies significantly reduced the heterogeneity of the included studies (I^2^ = 44%). Using FEM, a statistically significant age difference was found between the non-delayed discharge group and the delayed discharge group (MD = 1.33, 95% CI: 0.73–1.93, *p* < 0.0001), indicating that older patients may experience a prolonged postoperative recovery time and an increased risk of delayed discharge due to declining physical function and a greater burden of comorbidities. This finding emphasizes the importance of enhanced preoperative assessment and individualized postoperative care for elderly patients in clinical management, as shown in Figure 2.

**Figure 3:**
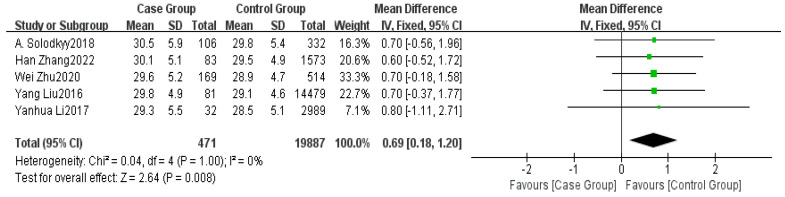
Forest plot of BMI [5,6,7,8,15].

Five studies provided data on BMI [5,6,7,8,15]. The meta-analysis indicated low heterogeneity among the studies (I^2^ = 0%). Using FEM, a significant difference in BMI was found between the non-delayed discharge group and the delayed discharge group (MD = 0.69, 95% CI: 0.18–1.20, *p* = 0.008), as detailed in Figure 3.

**Figure 4:**
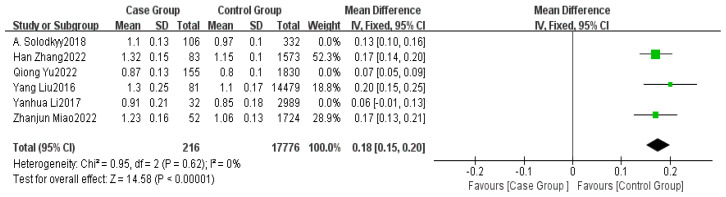
Forest plot of surgical duration [5,7,8,15,16,17].

Six studies provided data on surgical duration [5,7,8,15,16,17]. The meta-analysis indicated high heterogeneity among the studies (I^2^ = 89%). A sensitivity analysis was performed, and after removing three studies, the heterogeneity between the two groups significantly decreased (I^2^ = 0%). Using FEM, a significant difference in surgical duration was found between the non-delayed discharge group and the delayed discharge group (MD = 0.18, 95% CI: 0.15–0.20, *p* < 0.00001), as detailed in Figure 4.

**Figure 5:**
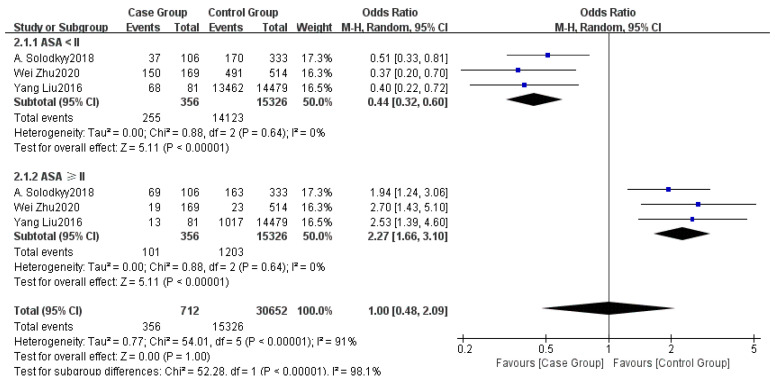
Forest plot of ASA [5,6,15].

Three studies provided data on the ASA classification [5,6,15]. Subgroup analysis indicated noticeable heterogeneity among the studies (I^2^ = 91%). Using a random effects model (REM), no significant difference was found in the ASA classification between the non-delayed discharge group and the delayed discharge group (OR = 1.00, 95% CI: 0.48–2.09, *p* = 1.00), as detailed in Figure 5.

**Figure 6:**
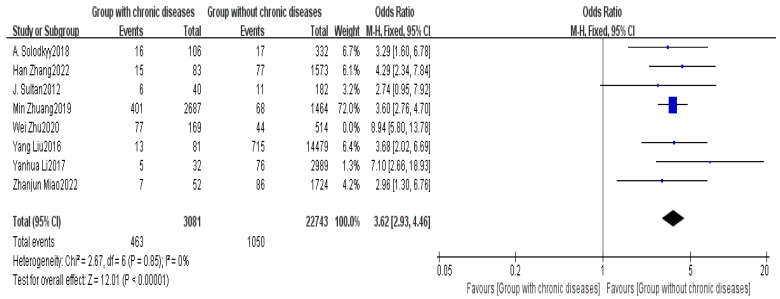
Forest plot of comorbid chronic diseases [2,5,6,7,8,14,15,16].

Eight studies provided data on comorbid chronic diseases [2,5,6,7,8,14,15,16]. The meta-analysis indicated noticeable heterogeneity among the studies (I^2^ = 57%). A sensitivity analysis was conducted, and after removing one study, the heterogeneity between the two groups significantly decreased (I^2^ = 0%). Using FEM, the results showed a significant difference in comorbid chronic diseases between the non-delayed discharge group and the delayed discharge group (OR = 3.62, 95% CI: 2.93–4.46, *p* < 0.00001), This highlights the need to pay close attention to patients with chronic diseases in clinical management, emphasizing enhanced preoperative evaluation and postoperative monitoring to reduce the incidence of delayed discharge, as detailed in Figure 6.

**Figure 7:**
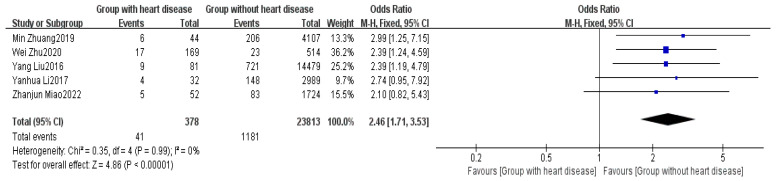
Forest plot of history of cardiovascular diseases [2,5,6,8,16].

Five studies provided data on the history of cardiovascular diseases [2,5,6,8,16]. The meta-analysis indicated low heterogeneity among the studies (I^2^ = 0%). Using FEM, a significant difference was found between the non-delayed discharge group and the delayed discharge group regarding the history of cardiovascular diseases (OR = 2.46, 95% CI: 1.71–3.53, *p* < 0.00001), as detailed in Figure 7.

**Figure 8:**
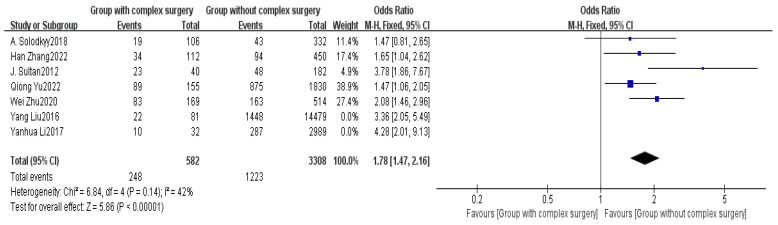
Forest plot of surgery complexity [5,6,7,8,14,15,17].

Seven studies provided data on the type of surgery [5,6,7,8,14,15,17]. The meta-analysis indicated high heterogeneity among the studies (I^2^ = 63%). A sensitivity analysis was performed, and after removing two studies, the heterogeneity between the two groups significantly decreased (I^2^ = 42%). Using FEM, the results showed a significant difference between the non-delayed discharge group and the delayed discharge group regarding the complexity of the surgery (OR = 1.78, 95% CI: 1.47–2.16, *p* < 0.00001), as detailed in Figure 8.

**Figure 9:**
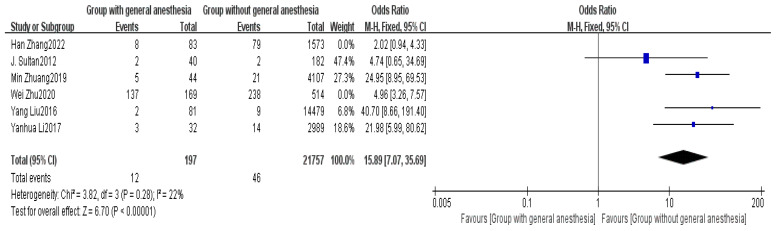
Forest plot of anesthesia type [2,5,6,7,8,14].

Six studies provided data on the type of anesthesia [2,5,6,7,8,14]. The meta-analysis initially indicated substantial heterogeneity among the studies (I^2^ = 82%), likely stemming from differences in chronic disease classification and incomplete data on anesthesia types across studies. A sensitivity analysis was conducted to address this variability, and after excluding studies contributing disproportionately to heterogeneity, the heterogeneity decreased significantly (I^2^ = 22%). This reduction indicates that the remaining studies provided more consistent data, enhancing the robustness of the overall findings. Using a fixed effects model (FEM), the results demonstrated a statistically significant difference between the non-delayed discharge group and the delayed discharge group regarding the type of anesthesia (OR = 15.89, 95% CI: 7.07–35.69, *p* < 0.00001). This finding highlights the clinical relevance of the anesthesia type, as general anesthesia may lead to delayed postoperative awakening, thereby increasing recovery time and the risk of delayed discharge. These results are detailed in Figure 9.

**Figure 10:**
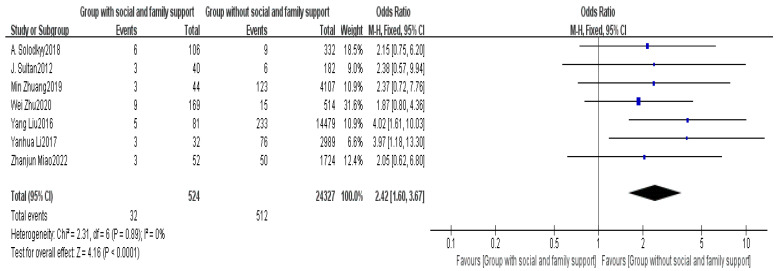
Forest plot of social family support [2,5,6,8,14,15,16].

Seven articles provided data on the lack of social family support [2,5,6,8,14,15,16]. The meta-analysis indicated a high level of heterogeneity among the studies (I^2^ = 0%). Using FEM, a significant difference was found in social family support between the non-delayed discharge group and the delayed discharge group (OR = 2.42, 95% CI: 1.60–3.67, *p* < 0.0001), as detailed in Figure 10.

**Figure 11:**
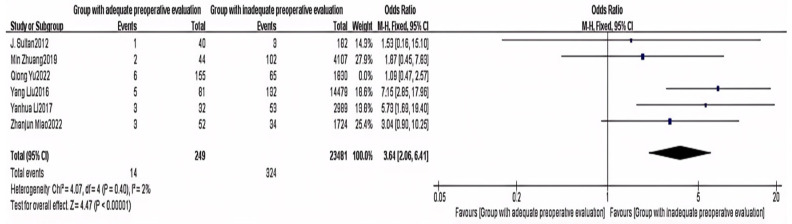
Forest plot of preoperative assessment [2,5,8,14,16,17].

Six articles provided data on preoperative assessment [2,5,8,14,16,17]. The meta-analysis indicated a high level of heterogeneity among the studies (I^2^ = 57%). A sensitivity analysis was conducted. After removing one study, the heterogeneity between the two groups significantly decreased (I^2^ = 2%). Using FEM, a statistically significant difference was found in preoperative assessment between the non-delayed discharge group and the delayed discharge group (OR = 3.64, 95% CI: 2.06–6.41, *p* < 0.00001), as detailed in Figure 11.

**Figure 12:**
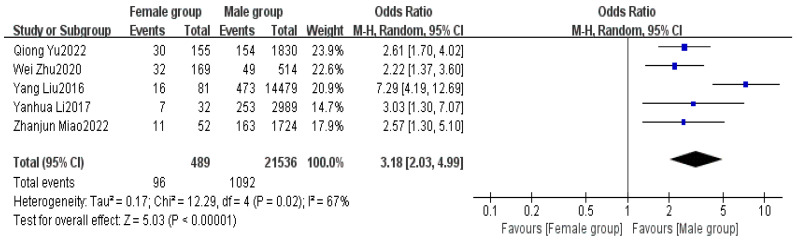
Forest plot of gender [5,6,8,16,17].

Five articles provided data on gender [5,6,8,16,17]. The meta-analysis indicated a high level of heterogeneity among the studies (I^2^ = 67%). Using REM, a significant difference was found in gender between the non-delayed discharge group and the delayed discharge group (OR = 3.18, 95% CI: 2.03–4.99, *p* < 0.00001). Male patients have a significantly higher risk of delayed discharge compared to female patients. This difference may be attributed to lower postoperative care compliance, inadequate pain management, or biases in the distribution of surgical types among male patients. This finding highlights the need for greater attention to postoperative management in male patients to reduce the risk of delayed discharge, as detailed in Figure 12.

**Figure 13:**
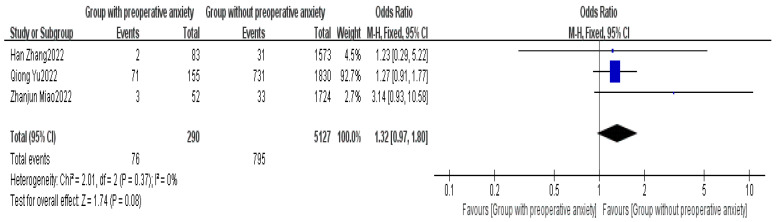
Forest plot of preoperative anxiety [7,16,17].

Three articles provided data on preoperative anxiety [7,16,17]. The meta-analysis indicated a high level of heterogeneity among the studies (I^2^ = 0%). Using FEM, no significant difference was found in preoperative anxiety between the non-delayed discharge group and the delayed discharge group (OR = 1.32, 95% CI: 0.97–1.80, *p* = 0.08), as detailed in Figure 13.

Some factors in the meta-analysis exhibited high heterogeneity, which was significantly reduced after sensitivity analysis and the exclusion of certain studies. The excluded studies, along with their authors, reasons for exclusion, and impact on the analysis, have been summarized to explain the reduction in heterogeneity, as detailed in Table 4.

## 4. Discussion

This study systematically assessed various preoperative risk factors for delayed discharge in day surgery patients through a meta-analysis, aiming to provide more effective evidence-based support for risk assessment and patient management in clinical practice. Day surgery is increasingly favored by hospitals and patients due to its advantages of shorter hospitalization time, lower costs, and a reduced risk of hospital-acquired infections [18]. However, the issue of delayed discharge remains one of the major challenges in the successful implementation of day surgery [19]. Exploring the impact of preoperative risk factors on delayed discharge is of significant importance, as identifying these factors can help optimize preoperative screening, improve surgical success rates, reduce unnecessary hospitalization time, lower healthcare costs, and enhance patients’ postoperative quality of life and satisfaction [20,21]. Additionally, through an in-depth analysis of these risk factors, physicians can take more targeted intervention measures to optimize the overall surgical process and patient recovery experience [21,22].

The results indicate that various factors have a significant impact on delayed discharge [23], including age, BMI, a history of chronic diseases, the type of anesthesia, a history of cardiac disease, gender, the duration of surgery, complex surgeries, a lack of social family support, and inadequate preoperative assessment [23,24,25]. Through a comprehensive analysis of these risk factors, we hope to provide more refined guidance for clinical practitioners, assisting them in making informed decisions in preoperative risk assessment and postoperative management.

The results of this study indicate that older patients face a higher risk of delayed discharge (MD = 1.33, *p* < 0.0001). This conclusion is consistent with previous research findings [26]. Older patients tend to have a slower postoperative recovery due to physical function decline, an increase in chronic diseases, and a diminished capacity for anesthetic drug metabolism [27]. Furthermore, common postoperative functional recovery issues in elderly patients, such as reduced muscle strength and balance, also increase the likelihood of delayed discharge [28]. Therefore, for elderly patients, clinicians should conduct a thorough preoperative assessment, such as utilizing the Barthel Index to predict recovery capacity, prioritizing minimally invasive techniques to reduce postoperative complications, and developing a personalized postoperative rehabilitation plan, including physical therapy and nursing interventions, to assist in the recovery of daily living abilities more quickly [29].

This study found that patients with a high BMI face a significant risk of delayed discharge (MD = 0.69, *p* = 0.008). Obesity not only increases the complexity of surgical procedures but also raises the risk of intraoperative and postoperative complications, such as respiratory and cardiovascular issues [30]. The increase in adipose tissue may affect the distribution and metabolism of anesthetic drugs, thereby prolonging recovery time [31]. Therefore, for patients with high BMI, preoperative weight management should be emphasized, and the choice of anesthesia should be optimized to reduce surgery-related risks. Additionally, enhancing monitoring and rehabilitation support for obese patients postoperatively can help improve their discharge speed.

Patients with comorbid chronic diseases, such as diabetes, hypertension, and chronic pulmonary diseases, also exhibited a significant risk of delayed discharge in this study (OR = 3.62, *p* < 0.00001). These chronic conditions can impair wound healing from surgical trauma and increase the likelihood of postoperative complications [32]. Diabetic patients, especially those with poor blood glucose control, may face a risk of delayed wound healing, while patients with cardiovascular diseases face a higher risk of cardiovascular events postoperatively [6]. Therefore, a thorough medical assessment and management of these high-risk patients must be conducted preoperatively to ensure the smooth progress of the surgical process and postoperative recovery. Preoperative optimization of medical conditions, such as strict glycemic control and cardiac function evaluation, should be performed. Postoperatively, close monitoring of vital signs and enhanced follow-up care are essential. Stabilization of the condition before surgery and close monitoring after surgery are essential measures to reduce the risk of delayed discharge for these patients.

The type of anesthesia also showed a significant impact in this study. Local anesthesia is more beneficial for postoperative recovery than general anesthesia, with fewer complications and shorter recovery times (OR = 15.89, *p* < 0.00001). This aligns with the rapid recovery requirements of day surgery [33]. Therefore, for surgical procedures suitable for local anesthesia, this method should be prioritized. In this study, local anesthesia includes regional anesthesia techniques such as spinal anesthesia and epidural anesthesia. The OR of 15.89 for general anesthesia underscores its strong association with delayed discharge, suggesting that regional anesthesia should be prioritized whenever feasible. This aligns with the goals of rapid recovery in day surgery and highlights the need for tailored anesthesia strategies based on patient characteristics and surgical requirements. Additionally, the surgical team should consider the patient’s physical condition, the complexity of the procedure, and postoperative recovery needs when developing the anesthesia plan to ensure optimal treatment outcomes and minimal risk [34].

A history of cardiovascular disease is another significant influencing factor (OR = 2.46, *p* < 0.00001). Surgery and anesthesia can place stress on cardiac function, resulting in a higher risk of cardiovascular events during and after surgery for patients with heart disease. To reduce the risk of postoperative complications in these patients, a thorough preoperative cardiac function assessment should be conducted, such as evaluating cardiac function status through electrocardiograms and echocardiograms [35]. During and after surgery, close monitoring of heart rate and blood pressure is essential, and for patients at risk of arrhythmias, antiarrhythmic medications should be administered promptly, along with enhanced monitoring [36].

The ASA classification is an important indicator for assessing patients’ preoperative health status [25]. This study found no significant impact of the ASA classification on delayed discharge (OR = 1.00, 95% CI: 0.48–2.09, *p* = 1.00), which may be attributed to the generally low ASA classifications and overall good health status of the majority of patients in this study. However, in the subgroup analysis, there were statistically significant differences between the subgroups of ASA scores < 2 and ASA scores ≥ 2 (*p* values < 0.00001). It is noteworthy that only three of the included studies reported ASA grades. Therefore, our findings may be biased due to the small sample size and limited number of studies. In other studies involving high-risk patients, the ASA classification often significantly predicts surgical outcomes [37]. Thus, clinicians should still emphasize the ASA classification in preoperative assessments to help identify potential high-risk patients and develop targeted surgical and anesthesia plans.

The impact of age on discharge time among day surgery patients varies significantly. The findings of this study indicate that elderly patients face a higher risk of delayed discharge (MD = 1.33, *p* < 0.0001). This observation aligns with conclusions from some studies, suggesting that advanced age is associated with prolonged postoperative recovery due to factors such as declining physical function, a higher prevalence of chronic diseases, and reduced metabolic capacity [38]. However, other studies argue that with optimized preoperative preparation and postoperative care, elderly patients may recover at a pace comparable to younger individuals, with no significant delays observed in certain types of surgeries [39]. These discrepancies may be attributed to factors such as the complexity of the surgery, preoperative interventions, and the overall health status of the patient. For example, minimally invasive procedures (e.g., ophthalmologic surgeries) appear less affected by age [40], whereas more complex or prolonged surgeries may significantly extend the postoperative recovery period for elderly patients. Additionally, the adequacy of preoperative assessments and postoperative support likely plays a critical role [41]. Future research should further investigate the mechanisms underlying the influence of age on recovery across different surgical types to optimize preoperative evaluations and develop personalized care strategies for elderly patients.

Obesity (high BMI) is commonly associated with delayed discharge. The results of this study reveal that patients with a higher BMI are at a significantly increased risk of delayed discharge (MD = 0.69, *p* = 0.008), consistent with most findings in the literature [42]. Obesity may prolong recovery by increasing intraoperative complexity and the risk of postoperative complications such as respiratory dysfunction and delayed wound healing [43]. However, some studies have reported no significant association between BMI and delayed discharge, which may be explained by several factors. The type of surgery can influence the sensitivity to BMI, with local anesthesia procedures being less affected by BMI compared to more complex surgeries [44]. Variability in the BMI classification criteria and sample distribution ranges across studies may also contribute to these inconsistencies [45]. Furthermore, adequate preoperative interventions may mitigate the negative impact of obesity on recovery to some extent [46]. Therefore, future studies should integrate surgical types and preoperative management to provide a more detailed analysis of postoperative recovery characteristics in obese patients. In clinical practice, enhancing preoperative optimization and providing personalized postoperative support for obese patients can improve recovery speed and reduce the risk of delayed discharge.

The duration of surgery and the complexity of the procedure are also important factors affecting discharge [19,47]. This study found that longer surgical times increase the likelihood of delayed discharge (MD = 0.18, *p* < 0.00001). An extended duration of surgery may increase intraoperative blood loss and delay the postoperative recovery process [48]. Therefore, the surgical team should focus on precise preoperative planning to minimize surgical time while enhancing the collaboration efficiency of the surgical team to reduce the impact of surgery duration on discharge.

Social family support plays a crucial role in patients’ postoperative recovery [49]. This study found that patients lacking social family support are more likely to experience delayed discharge (OR = 2.42, *p* < 0.0001). The presence and support of family members can help patients better cope with the challenges of postoperative recovery, particularly for elderly patients and those with reduced physical strength [50]. Therefore, assessing patients’ social family support during the preoperative evaluation and providing additional care resources for those lacking support, when necessary, is critical for ensuring a smooth postoperative discharge.

Preoperative anxiety is a common issue among patients. Although this study did not find a significant impact of preoperative anxiety on delayed discharge (OR = 1.32, 95% CI: 0.97–1.80, *p* = 0.08), some research suggests that preoperative anxiety may influence postoperative pain perception and the recovery process [51]. Anxious patients often lack confidence in the surgical process and postoperative recovery, which may lead to poorer postoperative compliance. Therefore, clinicians should assess patients’ psychological states preoperatively and implement psychological interventions, such as preoperative counseling, cognitive behavioral therapy (CBT), and relaxation training, to help alleviate anxiety and enhance postoperative recovery outcomes [52]. Preoperative anxiety is a significant psychological factor influencing postoperative recovery. This study recommends using the State-Trait Anxiety Inventory (STAI) as a standardized assessment tool. The STAI has demonstrated high validity and reliability, with its applicability and reliability verified in numerous clinical studies [53]. It provides a scientific basis for preoperative psychological evaluation and guides the development of personalized intervention strategies.

Gender differences also emerged as a factor affecting discharge in day surgery in this study (OR = 3.18, *p* < 0.00001). Male patients generally have a poorer postoperative recovery compared to females, likely due to less effective pain management and lower compliance with care among males [54]. To improve the postoperative recovery speed of male patients, health education should be strengthened, encouraging active participation in rehabilitation plans and providing more guidance and support in pain management. The OR of 3.18 for male patients indicates a higher risk of delayed discharge. Clinically, this suggests the importance of targeted postoperative interventions, including pain management and rehabilitation plans, to address this disparity.

This meta-analysis indicates that elderly patients, obese patients, those with chronic comorbidities, those with a history of heart disease, and those lacking social or family support are at a high preoperative risk and require special attention. For elderly patients (≥65 years), cardiac function assessment should not rely solely on age [55]. Instead, a comprehensive evaluation should consider the presence of comorbidities, such as hypertension or coronary artery disease, and current or past cardiac symptoms, including angina, dyspnea, and palpitations [56]. This multi-factorial approach ensures a more accurate risk stratification and guides the selection of appropriate preoperative tests. Building on this approach, patients with coronary artery disease or angina may require stress testing to assess ischemic risk [57], while those with dyspnea or suspected valvular disease should undergo echocardiography to evaluate ventricular function and valve integrity [58]. For palpitations or arrhythmic symptoms, Holter monitoring can help detect arrhythmias [59]. Tailored preoperative testing ensures diagnostic accuracy and improves perioperative safety. For suspected coronary artery disease, coronary CT angiography offers detailed visualization of coronary anatomy and stenosis [60]. In cases of suspected valvular disease with inconclusive transthoracic echocardiography, transesophageal echocardiography provides an enhanced assessment of valve structure and function [61]. These advanced tools refine risk stratification and inform perioperative planning. For these patients, a comprehensive preoperative evaluation and adequate preparation should be conducted to minimize surgical risks. Postoperatively, enhanced care and support are essential to promote recovery. For suitable surgical procedures, regional anesthesia should be prioritized to reduce the potential risks associated with general anesthesia. Additionally, efforts should be made to shorten the duration of the surgical procedure in order to minimize the occurrence of postoperative complications and ensure patient safety and recovery.

To enhance the clinical relevance of this study, key preoperative risk factors and their corresponding management strategies are summarized to guide clinical practice, as presented in Table 5.

Additionally, the Enhanced Recovery After Surgery (ERAS) program has been widely implemented across various surgical types, demonstrating significant efficacy in optimizing preoperative preparation and reducing hospital stay duration [62]. The management of preoperative time within the ERAS framework, including patient education, psychological interventions, and optimization measures, directly impacts postoperative recovery and the risk of complications [63]. This study did not systematically analyze the specific effects of preoperative time variations on delayed discharge, which may represent a limitation. Future research should further integrate the ERAS framework to explore the role of preoperative time optimization in surgical outcomes for high-risk patients, providing more evidence-based guidance for preoperative management in clinical practice.

The impact of surgical duration on postoperative outcomes may be influenced by various factors. This study did not classify surgical duration into detailed categories; however, existing evidence suggests that complex procedures, particularly those involving multiple anatomical systems, are typically associated with longer operative times and a positive correlation with delayed discharge risk [64]. Additionally, teaching hospitals may experience longer surgical durations due to training requirements [65], whereas surgeon experience and procedure frequency can significantly reduce operative times and improve postoperative outcomes [66]. Future research should conduct systematic analyses incorporating surgical types and key variables to more comprehensively evaluate the overall impact of surgical duration on postoperative recovery.

This study included a wide range of surgical types, from relatively simple procedures such as shoulder arthroscopy to more complex surgeries like gastrectomy. Differences in complexity and invasiveness among these procedures may influence postoperative recovery trajectories and the risk of delayed discharge. For example, complex open surgeries are typically associated with longer operative times and higher postoperative care demands [67], whereas minimally invasive procedures often enable faster recovery and reduce the risk of complications [68]. This heterogeneity in surgical types may limit the generalizability of the findings to specific surgical contexts. Future studies should consider stratified analyses based on the surgical approach and complexity to provide more precise insights into the risk factors for delayed discharge. Such stratified analyses would aid in optimizing preoperative risk assessment and developing postoperative care strategies tailored to different surgical types.

Limitations of this study: The number of included articles is limited, and most are retrospective studies, which may lead to retrospective bias. Additionally, there is considerable heterogeneity among the studies, potentially influenced by various factors such as patient characteristics, the types of surgery, and the anesthesia methods. Future research should aim to expand the sample size and include a greater variety of surgical types and patient populations, particularly with studies involving elderly patients and those with comorbid chronic diseases, to more comprehensively assess the impact of preoperative risk factors on discharge from day surgery. Furthermore, research on preoperative psychological states should be strengthened, especially regarding the influence of anxiety on surgical outcomes, to develop more effective intervention strategies that improve postoperative recovery and quality of life for patients. In this study, heterogeneity primarily arose from variations in patient characteristics and surgical types. To control for potential biases, we employed subgroup analysis and sensitivity analysis. These methods enhanced the explanatory power of key variables and the robustness of the study’s conclusions. Future research could incorporate more diverse patient populations through multi-center collaborations and optimize data standardization processes to ensure the generalizability and applicability of the results. Another limitation of this study is the absence of multivariable regression analysis. This restricts the ability to isolate the independent contribution of each risk factor while accounting for potential confounding variables. Although our findings provide valuable insights into the association between preoperative risk factors and delayed discharge, they do not fully elucidate the interactions or independent effects of these factors. Future studies should employ larger sample sizes, standardized data collection protocols, and advanced statistical methods, including multivariable models, to enhance the robustness and clinical applicability of the results. In addition, this study is limited by the lack of detailed subgroup data for certain populations and surgical types. Future research should involve larger and more diverse samples, as well as more refined data collection, to investigate these subgroups and reveal the subtle interactions between risk factors and delayed discharge outcomes.

## 5. Conclusions

This study systematically evaluated various preoperative risk factors affecting delayed discharge in day surgery patients, identifying that factors such as age, high BMI, comorbid chronic diseases, the type of anesthesia, a history of cardiovascular disease, gender, the duration of surgery, the complexity of the procedure, a lack of social family support, and insufficient preoperative assessment are closely associated with delayed discharge in patients. While these findings provide valuable evidence for enhancing preoperative risk assessments and patient management strategies, future research should address potential confounders and explore additional strategies to optimize surgical outcomes.

## Figures and Tables

**Table 1:** Basic characteristics of included studies.

Author	Country	Study Design	Type of Surgery	Data Collection Period	Data Source	Sample Size	Outcomes
J. Sultan 2012 [14]	UK	RCS	Arthroscopic Shoulder Surgery	2007–2008	SC	222	①②③⑤⑥⑦⑧
Yang Liu 2016 [5]	CHN	RCS	Cholecystectomy, Lower Extremity Varicose Vein Surgery, Gastrointestinal Polyp Resection	April 2012–August 2014	SC	14,560	①③④⑤⑥⑧⑨⑩⑪
Yanhua Li 2017 [8]	CHN	RCS	Inguinal Hernia Surgery, Cholecystectomy, Lower Extremity Varicose Vein Surgery	June 2012–June 2016	SC	3021	①③④⑤⑥⑦⑧⑨⑩⑪
A. Solodkyy 2018 [15]	UK	PCS	Laparoscopic Cholecystectomy	March 2010–October 2012	SC	500	①②③⑤⑦⑨⑩
Min Zhuang 2019 [2]	CHN	RCS	Cataract Surgery	August 2014–December 2016	SC	4151	①③④⑥⑦⑧
Wei Zhu 2020 [6]	CHN	RCCS	Digestive Endoscopy, Breast Surgery, Abdominal Surgery	May 2011–May 2019	SC	683	①②③④⑤⑥⑨⑪
Zhanjun Miao 2022 [16]	CHN	RCS	Mixed Hemorrhoid Surgery	January 2019–December 2021	SC	1776	①③④⑦⑧⑩⑪⑫
Qiong Yu 2022 [17]	CHN	RCS	Cholecystectomy, Lower Extremity Varicose Vein Surgery, Breast Mass Surgery, Gastrointestinal Polyp Resection	January 2016–December 2020	SC	1985	①⑤⑦⑧⑩⑪⑫
Han Zhang 2022 [7]	CHN	RCS	Cholecystectomy, Lower Extremity Varicose Vein Surgery, Gastrointestinal Polyp Resection, Inguinal Hernia Repair	April 2012–August 2014	SC	14,560	①③⑤⑥⑦⑨⑩⑫

UK, United Kingdom; CHN, China; PCS, prospective cohort study; RCS, retrospective cohort study; RCCS, retrospective case-control study; SC, single center. ① Age, ② ASA classification, ③ chronic comorbidities, ④ history of cardiac disease, ⑤ complex surgery, ⑥ type of anesthesia, ⑦ lack of social and family support, ⑧ preoperative assessment, ⑨ BMI, ⑩ duration of surgery, ⑪ gender, and ⑫ preoperative Anxiety.

**Table 2:** NOS scores.

Study	Study Design				Comparability	Exposure			Scores
	Case Definition	Case Representativeness	Selection of Controls	Definition of Controls	Comparability of Cases and Controls	Ascertainment of Exposure	Same Methods of Ascertainment for Cases and Controls	Non- Response Rate	
J. Sultan 2012 [14]	★	★	★	★	★	★	★		7 stars
Yang Liu 2016 [5]	★	★	★	★	★	★	★		7 stars
Yanhua Li 2017 [8]	★	★	★	★	★	★	★		7 stars
A. Solodkyy 2018 [15]	★	★	★	★	★	★	★		7 stars
Min Zhuang 2019 [2]	★	★	★	★	★	★			6 stars
Wei Zhu 2020 [6]	★	★	★	★	★	★	★		7 stars
Zhanjun Miao 2022 [16]	★	★	★	★	★	★	★		7 stars
Qiong Yu 2022 [17]	★	★	★	★	★	★	★		7 stars
Han Zhang 2022 [7]	★	★	★	★	★	★	★		7 stars

Rated 6–7 stars as high-quality literature.

**Table 3:** The key risk factors.

Key Risk Factors	OR/MD	95% CI	*p*-Value	I^2^
Age	MD = 1.33	0.73–1.93	<0.0001	68% → 44%
Surgical Duration	MD = 0.18	0.15–0.20	<0.00001	89% → 0%
Chronic Comorbidities	OR = 3.62	2.93–4.46	<0.00001	57% → 0%
Type of Anesthesia	OR = 15.89	7.07–35.69	<0.00001	82% → 22%
Gender (Male)	OR = 3.18	2.03–4.99	<0.00001	67%

The arrow indicates that sensitivity analysis significantly reduced heterogeneity, highlighting the excluded studies’ impact and enhancing result reliability.

**Table 4:** Excluded studies and their impact.

Author (Year)	Reason for Exclusion	Impact of Exclusion
A. Solodkyy (2018) [15]	Statistical model or method inconsistent with other studies	Affects analysis of age and surgical duration
Qiong Yu (2022) [17]	Different inclusion criteria with significant differences in patient population	Affects age, surgical duration, and preoperative evaluation analysis
Yanhua Li (2017) [8]	Key variables defined differently from other studies	Affects analysis of surgical duration and surgical type
Wei Zhu (2020) [6]	Classification of chronic diseases differs from other studies	Affects analysis of chronic comorbidities and type of anesthesia
Yang Liu (2016) [5]	Research objectives not aligned with the focus of this study	Affects analysis of surgical type
Han Zhang (2022) [7]	Did not provide complete stratified data on type of anesthesia	Affects analysis of type of anesthesia

**Table 5:** Evaluation and management strategies.

Characteristic Category	Risk Features	Evaluation Recommendations	Management Recommendations
Elderly Patients (≥65 years)	Multiple comorbidities and slower postoperative recovery.	Cardiac function assessment, taking into account comorbidities such as coronary artery disease and hypertension, as well as current or past symptoms including angina, dyspnea, and palpitations.	Develop a personalized postoperative rehabilitation plan: monitor vital signs and provide rehabilitation support.
	Potential cognitive decline.	Assessment of postoperative recovery ability (Barthel Index).	
Obese Patients (BMI ≥ 30)	Increased surgical complexity and risk of postoperative respiratory complications.	Pulmonary function testing and airway evaluation.	Preoperative weight management optimization; use of short-acting anesthetics; intraoperative monitoring of oxygen saturation.
	Delayed wound healing and abnormal metabolism of anesthetic drugs.	Assess diabetes and hypertension control.	
Patients with Chronic Comorbidities	Comorbidities increase the risk of postoperative complications and prolonged hospital stays.	Comprehensive evaluation of liver and kidney function.	Stabilize conditions preoperatively, adjust medications, and monitor vital signs intraoperatively.
	Conditions such as diabetes and cardiovascular diseases significantly impact postoperative recovery.	Evaluate blood glucose control and cardiac function status.	
Patients Lacking Social Support	Lack of postoperative support, leading to slower recovery progress.	Assess family support and postoperative care availability.	Provide community care services, extend hospital stay, and arrange follow-up calls.
Complex Surgeries	Prolonged operative time with higher risks of postoperative infection and organ dysfunction.	Conduct preoperative risk assessments; organize a multidisciplinary team (MDT).	Adopt a multidisciplinary collaboration model; enhance postoperative monitoring to prevent infection and thrombosis.
Choice of Anesthesia	General anesthesia is suitable for complex surgeries but may delay recovery.	Select an appropriate anesthesia method based on patient and surgical requirements (prioritize regional anesthesia).	Prioritize regional anesthesia; for complex surgeries, opt for short-acting general anesthesia supplemented with rapid recovery techniques.

## Data Availability

The data presented in this study are available on request from the corresponding author due to privacy.

## Abbreviations

MD = mean difference, CI = confidence interval, OR = odds ratio, *p* = significance probability.
